# Standardization of Light Scattering Measurement in Conjunction With Immunochemical Analysis

**DOI:** 10.6028/jres.080A.059

**Published:** 1976-08-01

**Authors:** Gregory J. Buffone

**Affiliations:** Department of Clinical Laboratories, Children’s Hospital National Medical Center and George Washington University, Washington, D.C. 20009

**Keywords:** Antibodies, antigens, biological polymers, immunochemical analysis, light scattering, polymers, proteins, solid standards

## Abstract

Light scattering methods for the physical analysis of synthetic and biological polymers necessitates the use of scattering standards and absolute light scattering measurements. Standardization has not been employed when light scattering has been used to monitor immunochemical reactions using a kinetic or thermodynamic mode.

The concentration of a specific protein present in a complex matrix such as urine, serum or cerebrospinal fluid, is measured by reacting the protein of interest with its specific antibody and then measuring the excess light scattering of the solution produced by the formation of antigen antibody complexes. The lack of established light scattering standards in the area of immunochemical measurements makes instrumental quality control difficult and has hindered direct comparison of data among investigators. Both solid and liquid light scattering standards would be necessary to encompass the wide range of instrumentation currently in use. Several solid standards which have been used in the past include reflecting diffusers such as vitrolite, magnesium carbonate crystals with a ground surface, magnesium oxide coatings on magnesium carbonate crystal, casein paint on vitrolite, and solid opal glass transmitting diffusers such as flashed opal glass and solid opal glass. These standards, while applicable to manual light scattering photometers, are not suitable for recently developed automated instrumentation. Liquid standards in the form of Ludox^®^, solutions of polystyrene, suspensions of small diameter latex spheres and even pure organic solvents could be used more easily with the continuous flow and discrete automated analyzers. The introduction of instrumental standards at this level of analysis would result in improved overall quality control and facilitate data and method comparison between laboratories.

## I. Introduction

Nephelometry, as applied to immunochemistry, measures the excess scatter caused by antigen antibody complexes formed in solution. The concentration of the unknown analyte is determined by use of appropriate calibration materials. Since only relative nephelometric measurements are required, absolute standardization of instrumentation has not been necessary for internal laboratory accuracy. However, while lack of standardization has not been deleterious in internal accuracy, comparison of data between laboratories and day to day internal laboratory instrumental quality control has been difficult at best. Poorly defined protein standards make the definition of a more standardized measuring system an acute problem.

## II. Application of Light Scattering to Immunochemistry

Quantitative immunochemical methods used since the 1930’s include immunoprecipitation in solution [1]^1^ and in a gel matrix [2]. Both approaches rely on the formation of a precipitate for the quantitation of the analyte. The methods are time consuming and are considered relatively tedious and expensive. The resurgence of nephelometric methods for the measurement of the extent of immunochemical reactions beginning in 1969 to date, can be traced to the improvement in efficiency and sensitivity provided by this approach.

### Manual and Flow Nephelometry

Fluorometers have been used almost exclusively to make 90° equilibrium light scattering measurements when following Immunochemical reactions. Numerous manual methods for the measurement of urine, serum and cerebrospinal fluid proteins have been reported [3–5]. Continuous flow systems have made automation of immunochemical reaction measurements feasible.

### Kinetic Light Scattering

The introduction of the centrifugal analyzer [6] has provided an automated instrument capable of precise kinetic measurement. The centrifugal analyzer has been applied to the automated kinetic measurement of antigen antibody reactions. Centrifugal analyzers have been used to measure scatter from antigen antibody complexes at 90° [7] and at angles between 15 and 30 degrees [8], with the smaller angles providing greater sensitivity due to the large complex size.

### Solid Phase Light Scattering

Recently Giaver [9] described the use of a solid phase protein preparation for the monitoring of immunochemical reactions. Protein or carbohydrate antigens were adsorbed onto a glass slide coated with small spheres of indium metal (~ 1000 Å in diameter). Incubation of the plates in an antiserum specific for the adsorbed antigen results in reaction between antigen and antibody causing a variation in the protein coat thickness on the metal spheres resulting in an increase in the amount of light scattered from areas of the plates at which a reaction has occurred. This technique can detect, by visual inspection, a reaction on a bovine albumin coated slide at an antibody concentration of 10^−4^g/1.

### Mie Scattering

Application of light scattering to the detection of rheumatoid factor provides a more sensitive and potentially quantitative means of measurement [10]. A homogeneous distribution of latex spheres whose particle diameter is approximately equal to the wavelength of light used for analysis, will produce characteristic maxima and minima intensities when scattering is observed at different angles. Any agglutination of human IgG coated spheres by rheumatoid factor will result in a loss of homogeneity of particle size and the characteristic maxima and minima intensity pattern, with changes in maxima slopes being related to the concentration of rheumatoid factor.

### Quasi-Elastic Light Scattering

The use of quasi-elastic light scattering holds the potential for measurement of antigen or antibody at a concentration of a few micrograms per liter, making this technique competitive with RIA in terms of sensitivity[11]. Broadening of laser spectral line widths caused by changes in the diffusion coefficient is used as an extremely sensitive means of detecting agglutination of antigen coated latex spheres.

## III. Instrumental Standardization

The diverse methods and instrumentation just reviewed would require a versatile material for instrumental standardization. Here I consider the types of standards that have been used and some of the most recent commercial materials marketed for calibration.

### Reflecting Diffusers

Several materials used as reflecting diffusers for measurement of primary beam intensity are outlined in table 1. These materials are useful for calibration of the instrument with respect to primary beam intensity, but do not provide the analogous calibration situation one encounters when using suspended scattering particles. Neutral filters for primary beam attenuation are usually necessary when using this approach. These filters represent an additional variable and possible source of error.

*Transmitting Diffusers, Flashed Opal Glass*, is simply a white glass fused with clear glass. The two types of glass are drawn simultaneously from their respective tanks and fused immediately as they cool. The drawing is done by mechanical means and results in a reproducibly smooth surface. *Opal Glass*, is blown white glass. Manual manipulation results in a more variable product with a more irregular surface than the flashed opal glass.

When dealing with reflecting diffusers, it should be noted that depending on the angle of measurement and the type of photo tube used, polarization effects caused by these diffusers can produce variation in the absolute intensity measured.

### Amorphous Solid Scattering Materials

Zimm [13] has described the preparation of a polystyrene methyl acrylate material and its use as a scattering standard. The material is prepared by adding 5 percent methyl acrylate to the polystyrene when the polymerization is half complete. The mixture results in an opalescent material suitable for scattering or turbidity measurements. Zimm observed that the turbidity measurements were temperature sensitive as would be expected for an amorphous solid. Zinc oxide suspended in an acrylic resin has also been used as a standard. This preparation is also susceptible to temperature fluctuation.

### Liquid Scattering Materials

Liquid turbidity standards have been used in industry for several years. The most commonly used material being a preparation known as Formazin [14]. Formazin, produced from a reaction between hydrazine sulfate and hexamethylenetetramine, is a suspension of relatively large particles which exhibit angular dependent light scattering and unsuitable sedimentation properties.

Other suspensions that have been used include silver iodide, silver sulfide, and barium sulfate, also known as McFarland’s standard. These suspensions show only transient stability and should be used with care.

Solutions of polystyrene prepared in organic solvents have been used as calibration material for scattering photometers, and provide a relatively stable standard when appropriately sealed and not subjected to excessive ultraviolet radiation.

An aqueous standard, in the form of highly cross linked spherical beads of styrene divinylbenzene, is available commercially from AMCO Service Company^2^ (4151 Middlefield Road, Palo Alta, CA 94303). Particle size ranges from 0.1 to 0.8 *μ*m. The suspension is reported to be temperature, time and pressure stable and to possess superior sedimentation characteristics relative to Formazin. Several advantages of the styrene divinylbenzene suspension make it attractive as a nephelometric standard: (1) the suspension can be infinitely diluted in an aqueous medium, (2) it can be accommodated in both manual and automated systems now in use for nephelometric or light scattering measurement and (3) it is nonreactive with glass vessels.

Ludox^®^ is a commercial colloidal suspension of silica marketed by the Dupont Chemical Corporation. Ludox has gained popularity in recent years for calibration purposes because of its ease in handling. There are several forms of Ludox,^®^ each having its own unique physical and chemical properties. Ludox^®^ stability depends on silica solid content, temperature, size and surface area of silica particles, pH, particle charge, and salt concentration and character. The specific effect of each parameter is dependent on the Ludox^®^ system under consideration. Ludox^®^ can react with glass vessels if not properly stabilized. A detailed discussion of the chemical properties can be found in the Dupont technical publication entitled, *Properties, Uses, Storage and Handling of Ludox Colloidal Silica.*

## IV. Applicability of Standardization

The following aspects of nephelometric immunochemical analysis would benefit directly from instrumental standardization.

### Quality Control

The antigen antibody reaction is intricate, producing antigen antibody complexes with different characteristics at different antigen antibody ratios. Quality control in essentially all clinical laboratories consists of the measurement of one or two reference sera or plasma per assay representing discrete ratios in restricted zones of the calibration curve. Instrumental standards would provide a means of monitoring results in all zones without resorting to multiple reference measurements in each set of analyses.

### Data Comparison

Currently the criteria for method acceptance consists of the evaluation of the clinical applicability of results generated by the method. Comparison cannot now be made on an analytical basis except by semiquantitative means due to the lack of reference methods and instrumental standardization.

### Calibrator and Reagent Evaluation

A reference scattering material is necessary to allow for evaluation of both calibrators and biological reagents. These materials can vary from source to source and lot to lot.

The brief discussion of variables intrinsic to immunochemical methodology demonstrates the applicability of an instrumental standard to provide a point of reference for improved overall quality control, method comparison and quality control of reagents and calibrators.

## V. Summary

Amorphous solid scatterers such as described by Zimm or liquid scatterers, such as a polystyrene solution or styrene divinylbenzene suspension, seem to be the most reliable and practical material for routine use based on the experience of this investigator.

Low molecular weight polystyrene samples (300–500,000 g/mol) dissolved in an organic solvent can serve as a scattering standard in both automated and manual systems and provide scattering independent of the angle of measurement. As mentioned earlier, scattering from the solutions can be relatively low and the sensitivity of the instrumentation should be considered. The stability of the individual scattering components with regard to temperature, pressure and also the sedimentation characteristics would be superior.

Styrene divinylbenzene and Ludox^®^ suspensions exhibit relatively high scatter even at 90° and can be diluted in an aqueous medium eliminating refractive index differences that might arise when comparing an organic solvent standard and an aqueous standard in an absolute measurement. Styrene divinylbenzene and Ludox^®^ need to be evaluated with respect to long term stability in reference to this type of application.

An amorphous solid scatterer made up of polystyrene and methyl acrylate or zinc oxide in an acrylic resin can provide a suitable standard for manual instrumentation if the temperature variation fluctuation is closely controlled.

Considerable work remains to be done with respect to the evaluation of long term stability and comparison of different light scattering standard materials before specific recommendations can be advanced.

## Figures and Tables

**Table I:** Reflecting diffusers [12]

Material	Description	*R*^a^
		
a. Vitrolite	White structural glass ground with #320 carborundum	0.816
b. MgCO_3_	Block scraped with straight edge	.964
c. MgO	Layer (0.5 mm) smoked onto MgCO_3_ from burning chips	.990
d. Casein paint #1	Commercial brand, painted on Vitrolite	.885

a Absolute reflectance

## Footnotes

1 Figures in brackets indicate the literature references at the end of this paper.

2 In order to adequately describe materials, it was occasionally necessary to identify the company and commercial products by name and trademark. In no instances does such identification imply endorsement by the National Bureau of Standards, nor does it imply that the particular product is necessarily the best available for the purpose.
